# Emergence of bla_NDM-5_ *Enterobacterales* in Swedish wastewater effluent

**DOI:** 10.1017/S0950268826101071

**Published:** 2026-01-30

**Authors:** Carolina Axelsson, Annie Justh de Neczpal, Åsa Sjöling, Ann-Sofi Rehnstam-Holm

**Affiliations:** 1 Bioanalysis, Kristianstad University Faculty of Natural Sciences, Sweden; 2 Centre for Antibiotic Resistance Reseach, University of Gothenburg Department of Chemistry and Molecular Biology, Sweden

**Keywords:** bla_NDM-5_, bla_OXA-181_, Carbapeneme resistant Enterobacterales, ST437, ST8346

## Abstract

Carbapenem-resistant Enterobacterales were isolated from the outlet of a wastewater treatment plant in Kristianstad in southern Sweden, during spring and summer of the year 2024. MALDI-ToF MS identification and subsequent whole-genome sequencing identified eight *Klebsiella pneumoniae* strains belonging to ST437 and ST873 and 10 *Escherichia coli* strains belonging to ST167, ST648, ST1284, and ST8346. All strains, except *K. pneumoniae* ST873, were NDM-5 positive. *K. pneumaniae* ST437 and *E. coli* ST8346 carried two carbapenemase genes, *bla*_NDM-5_ and *bla*_OXA-181_, as well as the extended-spectrum-β-lactamases (ESBL) gene *bla*_CTX-M-15_. These two multi-drug-resistant ST variants that are widespread globally, that have previously not been detected in clinical settings in Sweden, are now detected in treated wastewater in a Swedish middle-sized town.

*Klebsiella pneumoniae and Escherichia coli* are the most common carbapenem-resistant Enterobacterales (CRE), associated with the highest number of infections. Usually, they are normal inhabitants of the gut microbiota but can also be opportunistic pathogens that cause urinary tract infections, pneumonia, meningitis, surgical wound infections, and liver infections. Nosocomial, often deadly infections, include blood stream infections that can develop into sepsis [1].

CRE with resistance to last resort carbapenems are regarded as critical global health threats due to their pandemic spread and increasing mortality rates [2]. The aim of this study was to evaluate the sudden emergence of CRE that occurred in wastewater outlet samples collected in Kristianstad, Southern Sweden.

In spring (March) and summer (August) 2024, phenotypically CRE isolates were detected in samples from outlet water from the Kristianstad wastewater treatment plant (WWTP), which warranted a more intense sampling over 2 weeks in September–October 2024. The plant had the capacity to treat wastewater from 210,000 persons daily, wastewater from the city hospital and a large pig slaughterhouse during the studied period. Cleaning procedures included mechanical, biological and chemical processes. The treated effluent was thereafter pumped into the recipient lake Hammarsjön, which is an extended part of the river Helge Å (figure in Supplementary Materials). The samples were collected where the effluent from the plant is pumped out into the lake. Three litres of surface water were collected into 1 L sterilized glass bottles and transported back to the laboratory for further analyses within 2 h. Volumes of 300 ml x 3 from every flask were filtered through sterile 47 mm 0,2 μm filters (Supor^®^ 200, Pall Corporation, Michigan, USA) using a filtering devise equipped with a vacuum pump. The filters where thereafter placed onto KPC ChromAgar™ (Paris, France) plates and incubated for 18–24 h at 37 °C. In total 55 CRE isolates were obtained from these samples subsequently identified as *E. coli* (*n* = 29) and *K. pneumoniae* (*n* = 26) by Microflex Biotyper MALDI-ToF MS (Bruker, Bremen, Germany) using the 1,829,023 Maldi Biotyper Compass Library. Total counts of bacteria were not performed. PCR analyses were achieved with primers and amplification protocols as described by Mentasti *et al.* (2019) using SYBRGreen and melt curve analyses [3]. The analyses indicated *bla*_NDM_ genes in 94% (52/55) and *bla*_OXA-48_-like genes in 56% (31/55) of the isolates (data not shown). Phenotypical antibiotic resistance to meropenem were detected by the standard disc diffusion method according to European Committee on Antimicrobial Susceptibility Testing (EUCAST, https://www.eucast.org/bacteria/methodology-and-instructions/disk-diffusion-and-quality-control/).

To further characterize the isolates, 18 representative isolates, ten *E. coli* and eight *K. pneumoniae*, were whole genome sequenced. The selection of the isolates was based on different dates of isolation and species. DNA was prepared using DNAeasy Blood and Tissue kit from Qiagen (Hilden, Germany) and sequenced at Eurofins Genomics (Constance, Germany) using INVIEW Resequencing of Bacteria on Illumina NovaSeq X+, PE150 mode. The isolates were analysed for multi-locus sequencing type, plasmid type, virulence genes, and antibiotic resistance genes (ARGs) [4]. The phylogenetic tree in Figure 1 shows how the two species group separately, with each species comprising different sequence types (STs) that exhibited conserved plasmid profiles, and where all isolates were multi-drug-resistant (MDR). MDR is defined here as presence of ARGs providing resistance to ≥3 antibiotic classes.

**Figure 1.**
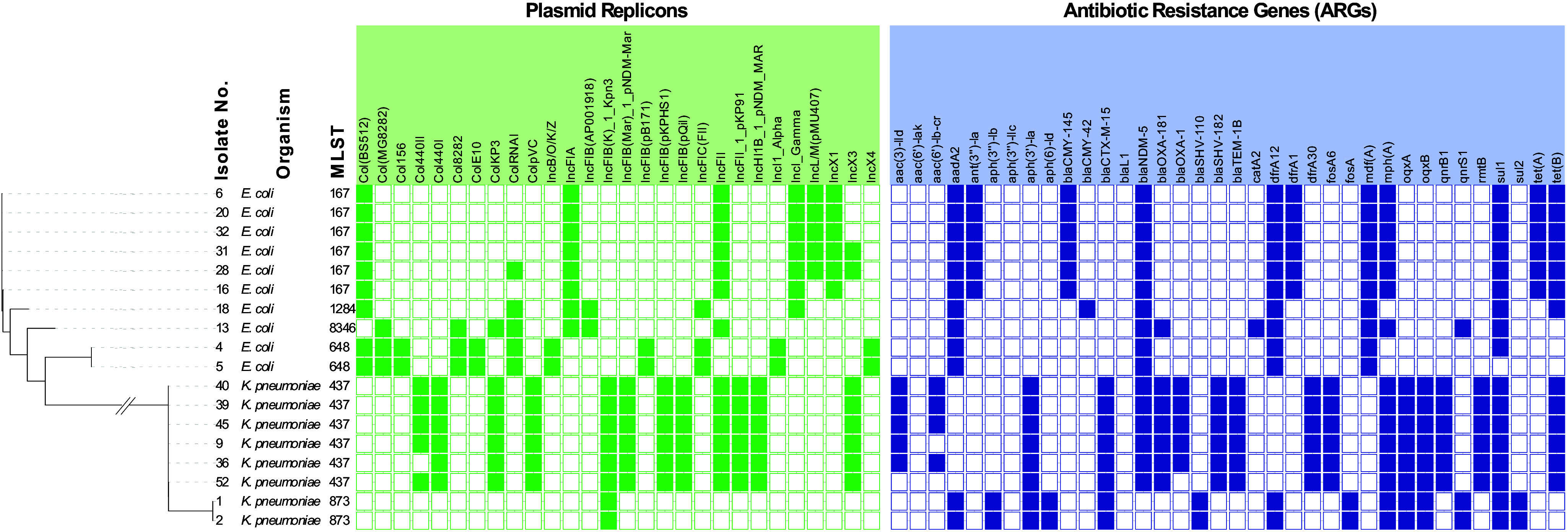
Phylogenetic tree of all isolates, showing isolate number, species, multi locus sequencing types (MLST), plasmid replicons (Inc groups), and presence of antibiotic resistance genes (ARGs). The sequencing results were analysed using BACTpipe, a pipeline for WGS bacterial genomes [3]. In the pipeline, fastp is used for pre-processing of paired ends, followed by Kraken2 for taxonomic identification and shovill for *de novo* assembly. Lastly, genome annotation is completed using prokka and statistics about the assembly and annotation are reported by MultiQC. MLST was determined using the achtman scheme (mlst v. 2.23.0), plasmid types were determined using ABRicate PlasmidFinder (2014), virulence genes were identified using ABRicate VFDB (2016) and ARGs were identified using ABRicate ResFinder (v. 4.0, 2020). Phylogenetic analyses were carried out using Mashtree (2022) where the final phylogenetic tree annotations were produced using iTOL (v. 7.0, https://itol.embl.de/). Sequence data are available under GenBank accession numbers JBRMLF000000000 to JBRMLW000000000.

The *E. coli* isolates belonged to ST167 (*n* = 6), ST648 (*n* = 2), ST1284 (*n* = 1), and ST8346 (*n* = 1). The *K. pneumoniae* isolates belonged to ST437 (*n* = 6) and ST873 (*n* = 2). Analysis with ResFinder (https://genepi.food.dtu.dk/resfinder) revealed NDM-5 metallo-β-lactamase genes in all *E. coli* and in the *K. pneumoniae* ST437 isolates. The isolates that contained both *bla*_NDM-5_ and *bla*_OXA-181_ belonged to *K. pneumoniae* ST437 and *E. coli* ST8346 (see supplementary material). *E. coli* ST167 and ST1284 were PCR positive for *bla*_OXA-48_ but no corresponding genes were detected by ResFinder.

Virulence genes were detected in all isolates. *K. pneumoniae* are divided into classic (cKp) and hypervirulent *K. pneumoniae* (hvKp), where the latter is characterized by a hypermucoid phenotype and presence of iron acquisition siderophores, including yersiniabactin, salmochelin, aerobactin, and enterobactin [5]. The yersiniabactin operon encoded by the *ybtAEPQSTUX operon, irp1 and irp2*, and siderophore receptor *fyuA* were found in all ST437. Presence of the aerobactin receptor gene (*iutA)* and aerobactin synthesis *operon (iucABCD*) is considered a hallmark of hvKp, enabling growth in iron-poor host environments. However, the *K. pneumoniae* detected in this study lacked both the *rmpA/rmpA2* locus conferring a hypermucoviscous phenotype and the full aerobactin and salmochelin loci and are thus not hvKp.

The two *K. pneumoniae* isolates from March belonged to ST873, a type previously known to carry *bla*_NDM-1_. Interestingly, these isolates were tested intermediate and resistant to meropenem, respectively, by the disc diffusion method, but harboured only the extended-spectrum β-lactamases *bla*_SHV-110_ and *bla*_CTX-M-15_. Therefore, the underlying mechanism of carbapenem resistance in the ST873 isolates requires further investigation.

*E. coli* ST167 and ST1284 both belong to globally dominant CRE *E. coli* types. In this study, ST1284 harboured the aerobactin operon, whereas ST167 was negative for this operon but contained the yersiniabactin operon. This study also detected ST8346, an emerging multi-resistant CRE type with recently reported co-carriage of *bla*_NDM-5_ and *bla*_OXA-181_ on IncF and IncX plasmids, respectively [6]. Finally, ST648 is also an emerging CRE clone. Thus, all the *E. coli* types identified in the WWTP outlet water are well established pathogens implicated in urinary tract infections, bloodstream infections, sepsis, and wound infections [6].

The development of MDR in *K. pneumoniae* and *E. coli* may result in treatment failures and is a global health problem. Particularly, the recent emergence of high carbapenemase resistance is a cause of concern [1]. In Sweden, mandatory laboratory reporting of CRE has been practiced since 2007. Reports from 2024 show an incidence of CRE infections of 3.9 cases per 100,000 inhabitants [7]. The most frequent sequence types reported were *K. pneumoniae* ST147, ST307, and ST395, and for *E. coli* ST167, ST69, and ST648, while *bla*_OXA-48_ followed by *bla*_NDM_ were the most common carbapenemases. In this study, we determined that several isolates recovered over a 2-week period were *K. pneumoniae* ST437. This type belongs to the clonal complex CC11 and is considered an emerging clone with epidemic potential. Additionally, it is frequently found in nosocomial infections in humans, as well as in environmental sources such as rivers and wastewater in many parts of the world [8]. NDM-5-positive *E. coli* has increased during the last years in Europe, and *E. coli* ST167 is predominant [9].

Several *K. pneumoniae* and *E. coli* STs associated with carbapenemases and/or ESBL carriage, seem to accumulate in wastewater and sewage and are often recovered from inlets to WWTPs [10]. Hence, WWTP inlets can provide a reflection on the carrier status of larger populations in communities [11], while detection of multidrug-resistant and virulent bacteria in treated effluent from WWTPs, as in the case of the isolates analysed in this study, indicates a high contribution from incoming water with increasing risk of resistance transmission [12].

The temporarily detection of *K. pneumoniae* ST437 and *E. coli* ST167 in the treated effluent from the WWTP in Kristianstad community was preceded by over 10 years (2014–2024) of surveillance sampling, where CRE was never isolated. The presence of *bla*_NDM-5_ in the Swedish environment is thus of great concern and suggests that highly virulent and multidrug-resistant clones may be more common in Sweden than previously known.

## Supporting information

10.1017/S0950268826101071.sm001 Axelsson et al. supplementary material Axelsson et al. supplementary material

## Data Availability

The data that support the findings of this study are openly available at GenBank (https://www.ncbi.nlm.nih.gov/genbank/) reference numbers JBRMLF000000000 to JBRMLW000000000.

## Supplementary material

The supplementary material for this article can be found at http://doi.org/10.1017/S0950268826101071.
