# Supplementary material for: Emergence of blaNDM-5 Enterobacterales in Swedish wastewater effluent
Source: Epidemiol Infect. 2026 Jan 30;154:e23. doi: 10.1017/S0950268826101071 (PMC12914470; doi:10.1017/S0950268826101071)
Supplement: Axelsson et al. supplementary material [file S0950268826101071sup001.zip › Table 1 WGS data 260110.docx]

Table 1. Isolates collected in 2024 with carbapenem-resistance and corresponding WGS data.

| **Isolate** | **Species** | **Date** | **ST** | **Accession number**  **(GenBank)** | **Carba-penemas genes** | **Plasmid replicons** | **Virulence genes** |
| --- | --- | --- | --- | --- | --- | --- | --- |
| 1 | *K. pn* | 240305 | ST873 | JBRMLW  000000000 | None | IncFIB(K) (Kpn3); IncFII | *iutA*; *fimH*; *mrkD*; *entS;* *upaG* |
| 2 | *K. pn* | 240305 | ST873 | JBRMLV  000000000 | None | IncFIB(K) (Kpn3); IncFII | *iutA*; *fimH*; *mrkD*; *entS*; *upaG* |
| 9 | *K. pn* | 240923 | ST437 | JBRMLP  000000000 | *bla*_NDM-5_, *bla*_OXA-181_ | IncX3; ColKP3; IncHI1B(pNDM-Mar); IncFIB(Mar); IncFIB(K) (Kpn3); IncFIB(pQil); IncFII | *fyuA*; *iutA*; *fimH*; *mrkD*; *ent*; *ecpA* |
| 36 | *K. pn* | 241003 | ST437 | JBRMLJ  000000000 | *bla*_NDM-5_, *bla*_OXA_*_-181_* | IncX3; ColKP3; IncHI1B(pNDM-Mar); IncFIB(Mar); IncFIB(K) (Kpn3); IncFIB(pQil); IncFII | *fyuA*; *iutA*; *fimH*; *mrkD*; *ent*; *ecpA* |
| 39 | *K. pn* | 241003 | ST437 | JBRMLI  000000000 | *bla*_NDM-5_, *bla*_OXA-181_ | IncX3; ColKP3; IncHI1B(pNDM-Mar); IncFIB(Mar); IncFIB(K) (Kpn3); IncFIB(pQil); IncFII | *fyuA*; *iutA*; *fimH; mrkD*; *ent*; *ecpA* |
| 40 | *K. pn* | 241003 | ST437 | JBRMLH  000000000 | *bla*_NDM-5_, *bla*_OXA-181_ | IncX3; ColKP3; IncHI1B(pNDM-Mar); IncFIB(Mar); IncFIB(K) (Kpn3); IncFIB(pQil); IncFII | *fyuA*; *iutA*; *fimH; mrkD*; *ent*; *ecpA* |
| 45 | *K. pn* | 241003 | ST437 | JBRMLG  000000000 | *bla*_NDM-5_, *bla*_OXA-181_ | IncX3; ColKP3; IncHI1B(pNDM-Mar); IncFIB(Mar); IncFIB(K) (Kpn3); IncFIB(pQil); IncFII | *fyuA*; *iutA*; *fimH; mrkD*; *ent*; *ecpA* |
| 52 | *K. pn* | 241003 | ST437 | JBRMLF  000000000 | *bla*_NDM-5_, *bla*_OXA-181_ | IncX3; ColKP3; IncHI1B(pNDM-Mar); IncFIB(Mar); IncFIB(K) (Kpn3); IncFIB(pQil); IncFII | *fyuA*; *iutA*; *fimH; mrkD*; *ent*; *ecpA* |
| 4 | *E. coli* | 240305 | ST648 | JBRMLU  000000000 | *bla*_NDM-5_ | IncB/O/K/Z; IncFIB(pB171); IncFIC(FII); IncI1-Alpha; IncX4; multiple Col plasmids | *fyuA*; *iutA*; *papC*; *fimH*; *upaG*; *lpfA* |
| 5 | *E. coli* | 240305 | ST648 | JBRMLT  000000000 | *bla*_NDM-5_ | IncB/O/K/Z; IncFIB(pB171); IncFIC(FII); IncI1-Alpha; IncX4; multiple Col plasmids | *fyuA*; *iutA*; *papC*; *fimH*; *upaG*; *lpfA* |
| 6 | *E. coli* | 240910 | ST167 | JBRMLS000000000 | *bla*_NDM-5_ | IncFIA; IncFII; IncI-Gamma; IncX1; Col(BS512) | *fyuA*; *iutA*; *fimH*; *mrkD*; *papC*; *usp* |
| 16 | *E. coli* | 240923 | ST167 | JBRMLP  000000000 | *bla*_NDM-5_ | IncFIA; IncFII; IncI-Gamma; IncX1; Col(BS512) | *fyuA*; *iutA; fimH;* *mrkD*; *papC*; *usp* |
| 20 | *E. coli* | 240923 | ST167 | JBRMLN  000000000 | *bla*_NDM-5_ | IncFIA; IncFII; IncI-Gamma; IncX1; Col(BS512) | *fyuA*; *iutA*; *fimH*; *mrkD*; *papC*; *usp* |
| 31 | *E. coli* | 241003 | ST167 | JBRMLL000000000 | *bla*_NDM-5_ | IncFIA; IncFII; IncI-Gamma; IncX1; Col(BS512) | *fyuA*; *iutA*; *fimH;* *mrkD;* *papC*; *usp* |
| 32 | *E. coli* | 241003 | ST167 | JBRMLK  000000000 | *bla*_NDM-5_ | IncFIA; IncFII; IncI-Gamma; IncX1; Col(BS512) | *fyuA*; *iutA*; *fimH; mrkD;* *papC*; *usp* |
| 28 | *E. coli* | 241003 | ST167 | JBRMLM  000000000 | *bla*_NDM-5_ | IncFIA; IncFII; IncI-Gamma; IncX1; Col(BS512); IncX3; IncL/M(pMU407) | *fyuA*; *iutA*; *fimH;* *mrkD;* *papC*; *usp* |
| 13 | *E. coli* | 240923 | ST8346 | JBRMLQ  000000000 | *bla*N_DM-5_, *bla*_OXA-181_ | IncX3; ColKP3; IncFII; IncFIA; Col(MG828) | *fyuA*; p*apC*; e*cpA* |
| 18 | *E. coli* | 240923 | ST1284 | JBRMLO  000000000 | *bla*_NDM-5_ | IncFIA; IncFIB(AP001918); IncFIC(FII); IncI-Gamma; ColRNAI; ColBS512; (likely IncX3) | *iutA*; *iucC*; *fimH* |
