# Supplementary figures and images for: Emergence of blaNDM-5 Enterobacterales in Swedish wastewater effluent
Source: Epidemiol Infect. 2026 Jan 30;154:e23. doi: 10.1017/S0950268826101071 (PMC12914470; doi:10.1017/S0950268826101071)

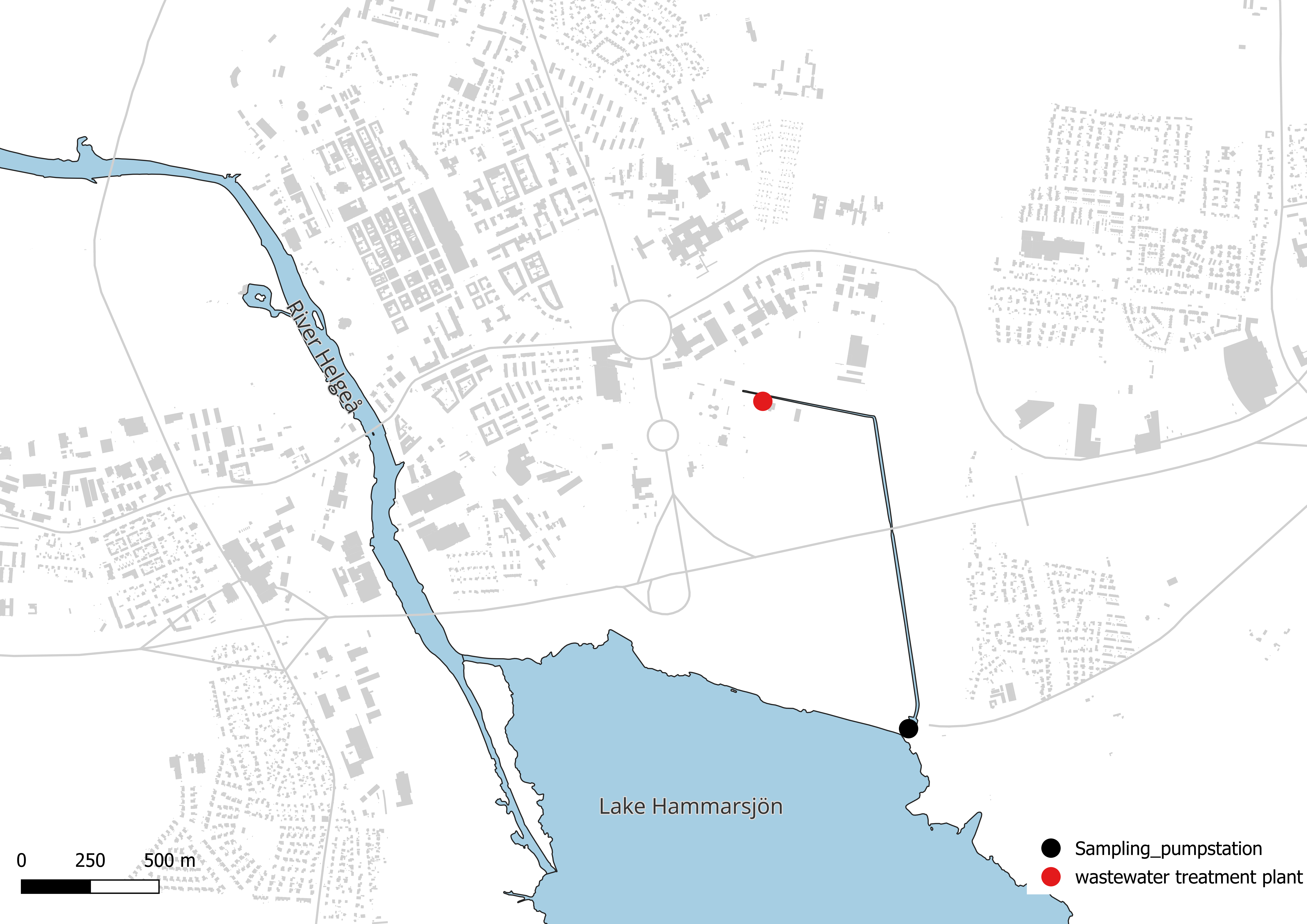

Supplement: Axelsson et al. supplementary material [file S0950268826101071sup001.zip › Map of WWTP Kristianstad.png]
